# The hot sites of α-synuclein in amyloid fibril formation

**DOI:** 10.1038/s41598-020-68887-2

**Published:** 2020-07-22

**Authors:** Anahita Khammari, Seyed Shahriar Arab, Mohammad Reza Ejtehadi

**Affiliations:** 1 Department of Biophysics, School of Biological Sciences, Tarbiat Modares University, Tehran, Iran; 2 Physics Department, Sharif University of Technology, P.O. Box 11155-9161, Tehran, Iran; 3 School of Nano-Science, Institute for Research in Fundamental Sciences (IPM), Tehran, Iran

**Keywords:** Computational biophysics, Intrinsically disordered proteins, Biophysics, Computational biology and bioinformatics, Structural biology

## Abstract

The role of alpha-synuclein (αS) amyloid fibrillation has been recognized in various neurological diseases including Parkinson’s Disease (PD). In early stages, fibrillation occurs by the structural transition from helix to extended states in monomeric αS followed by the formation of beta-sheets. This alpha-helix to beta-sheet transition (αβT) speeds up the formation of amyloid fibrils through the formation of unstable and temporary configurations of the αS. In this study, the most important regions that act as initiating nuclei and make unstable the initial configuration were identified based on sequence and structural information. In this regard, a Targeted Molecular Dynamics (TMD) simulation was employed using explicit solvent models under physiological conditions. Identified regions are those that are in the early steps of structural opening. The trajectory was clustered the structures characterized the intermediate states. The findings of this study would help us to better understanding of the mechanism of amyloid fibril formation.

## Introduction

Aggregation of proteins into amyloid fibrils is associated with many neurological diseases such as Alzheimer’s disease (AD)^1^, Parkinson’s disease (PD)^2^, and Type-2 Diabetes (T2D)^3^. PD is identified as the second most common neurodegenerative disorder and about 7 million people over 60 years old are estimated to suffer from this disorder^4^. Structural dysfunction of αS and self-assembly of αS into toxic oligomers and fibril species are the most important reasons for the development of PD^5^. The αS is among Intrinsically Disordered Proteins (IDPs), which is abundant in the human brain encoded by an *SNCA* gene located on chromosome 4^6,7^. Protein structure is divided into three parts: (i) Amphipathic N-terminal region (1–60 residues), (ii) Non-amyloid-β component (NAC), and iii) Acidic and proline-rich region having no regular structure (C-terminal segment). The αS propensity to aggregation and fibrils formation causes the conformational change from disordered monomers into dimers, oligomers and then protofibrils (premature fibrils)^8^.

Many therapeutic approaches of PD are based on the prevention of amyloid fibrillation or destabilization of pre-existing fibrils^9,10^. Among them, the approaches which only have focused on stabilization of protein folding, binding blocking of neuron membrane or protein immunotherapy have not been clinically successful^4,11^ and PD treatment has remained a challenging topic^12^. Some emerging therapeutic methods are based on the design of peptides against different parts of αS, which have been reported to have more effective therapeutic results through inhibition of the oligomers (or fibril) formation and blocking of αS aggregation^13^. Designed peptides are randomly selected from different parts of the protein and are tested for their efficiency. Finding efficient therapeutic peptides by random scanning method has been time-consuming over the last decades. In the rational procedure, the most important regions of protein that play a key role in protein deformation mechanism are identified and therapeutic agents are designed based on these regions.

The extreme compatibility of αS causes the protein to have different states whose molecular mechanism of evolution and their relationship are unknown^14^. Different inherently disordered^15,16^, helical^17^, or a combination of the two^18^ are described for α-syn. In a reversible binding to the membrane, αS can bind the membrane upon the structural transition from a random coil to α-helix. It should be noted, that the fibril formation process can be separately performed from helical and random coil structures in vivo^19^ but Meade et al*.* stated that because of the larger population of helix-rich structures in the presence of the membrane, the helical state can be presented as a functional state of the protein^20^. Indeed, there are some evidence that helical αS monomers play important roles in both intracellular and extracellular fibrillation mechanisms through the formation β-sheet-rich structures^14,21^. To closely examine the structural deformation, we focus on protein monomer. At the early stages of the αS fibrillation process, a conformational transition occurs from helical to extended structures followed by the creation of beta-sheets, and eventually forming of amyloid fibrils^22^. Multibiological events, either environmental or genetics can lead to the occurrence of this conformational transition consequently resulting in loss of normal function of the protein by disrupting the function of mitochondria and degradation of the membrane^23,24^. Critical sites of αS influencing on β-formation at early stages can be identified by focusing on the α-β conformational transition (αβT).

Since many conformational transitions occur at time scales longer than a few microseconds, enhanced sampling methods have been developed to explore the appropriate phase space to solve some of the problems in proteins^25^. Targeted Molecular Dynamics^26^ is an approach that depends on a target structure that can induce conformational changes to the known target structure using a time-dependent geometrical constraint at biological temperature. This method is suitable for transitions of protein structure between two specified conformational states such as αβTs in amyloid fibrillation. In transitions, the system is independently enforced the height of energy barriers, while structural dynamics are only minimally influenced by the RMSD constraint and can explore configurational space for finding transition states. This helps us to find critical sites in αβT at biological temperatures. These regions will be identified as the most changeable sites in the transition from helix to extended structures in the αS single chain, and they are the main cores in the formation of the amyloid fibrils. Knowing about the structure and sequencing information of these regions can be useful to understand the mechanism of αβT of the αS and help us to design a better generation of amyloidogenic peptides against PD. Besides, this study can be a suitable model for the detection of β-forming regions in other aggregation-prone proteins.

## Results and discussion

### The hot sites of αS

**Figure 1:**
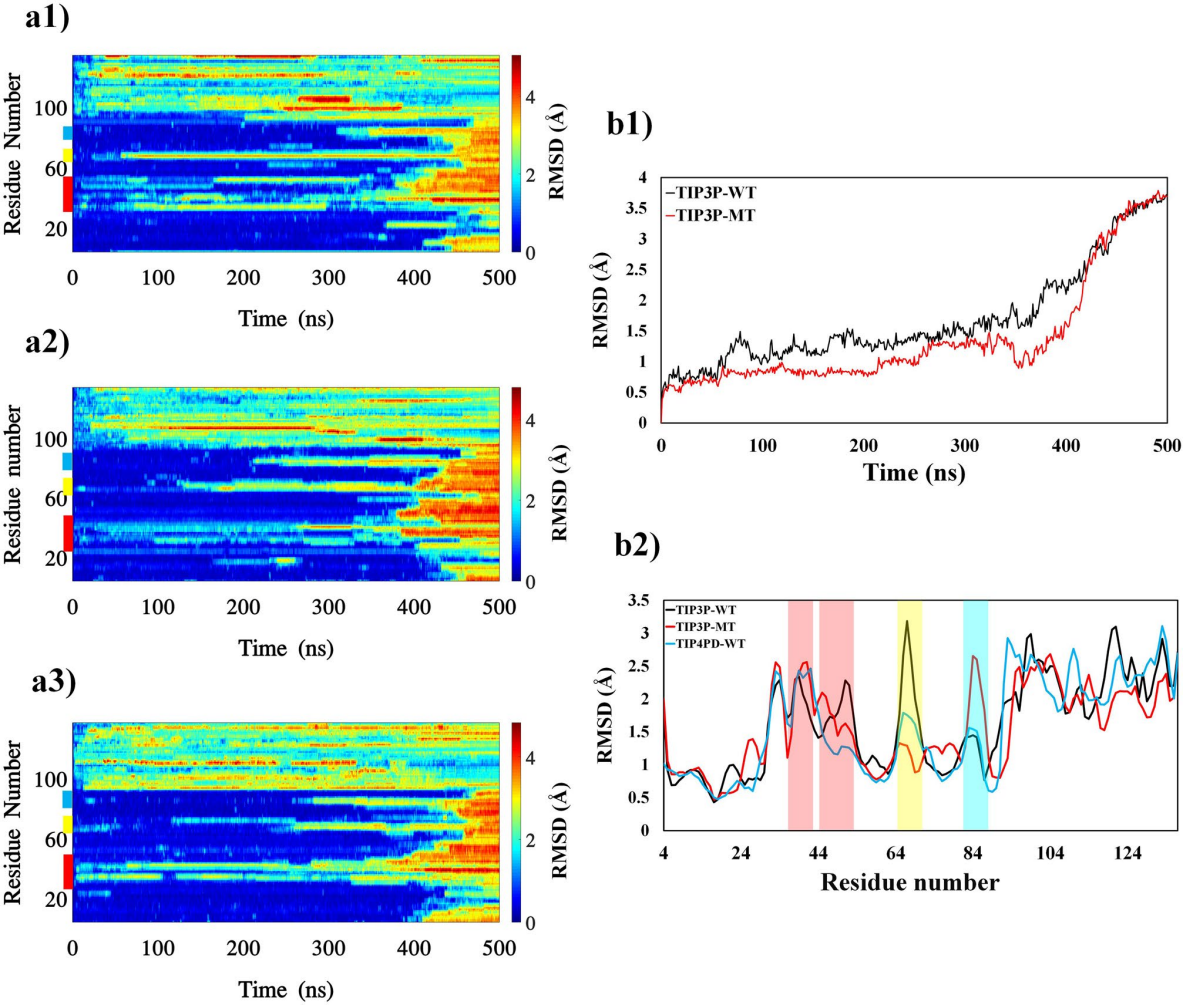
The RMSD of protein sections during the TMD simulations. The figures in left panels show kymographs of three sets of the TMD simulations, protein wild type in TIP3P water model (TIP3P-WT), mutated protein in TIP3P water model (TIP3P-MT) and protein wild type in TIP4PD water model (TIP4PD-WT). Color bar in the right side indicates the values and the locations of the hot sites are colored as red, yellow and cyan in the left side of the plots indicating the first, second and third priorities, respectively. B1 compares the average RMSD for valine and alanine residues in the hot sites (see text). B2 compares time averaged RMSD of the residues for the three sets of the simulations.

**Figure 2:**
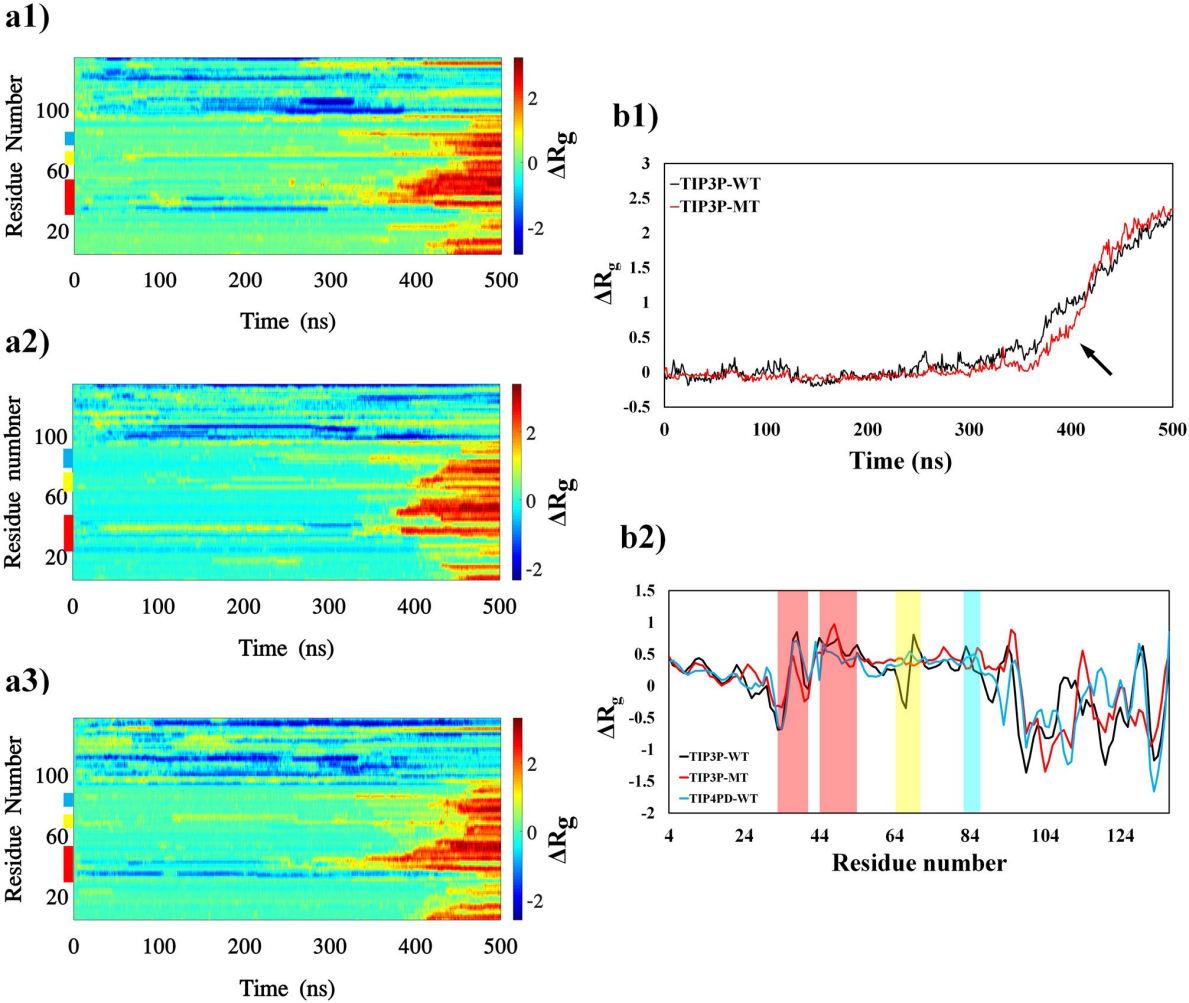
The difference between gyration radius ($\Delta {R}_{g}$) of each frame configurations with the first frame during TMD trajectories. The figures in left panels show kymographs of three sets of the TMD simulations, protein wild type in TIP3P water model (TIP3P-WT), mutated protein in TIP3P water model (TIP3P-MT) and protein wild type in TIP4PD water model (TIP4PD-WT). Color bar in the right side indicates the values and the locations of the hot sites are colored as red, yellow and cyan in the left side of the plots indicating the first, second and third priorities, respectively. B1 compares the average $\Delta {R}_{g}$ for valine and alanine residues in the hot sites (see text) the black arrows point to the most different of $\Delta {R}_{g}$ between the valine and alanine residues. B2 compares time averaged $\Delta {R}_{g}$ of the residues for the three sets of the simulations.

**Figure 3:**
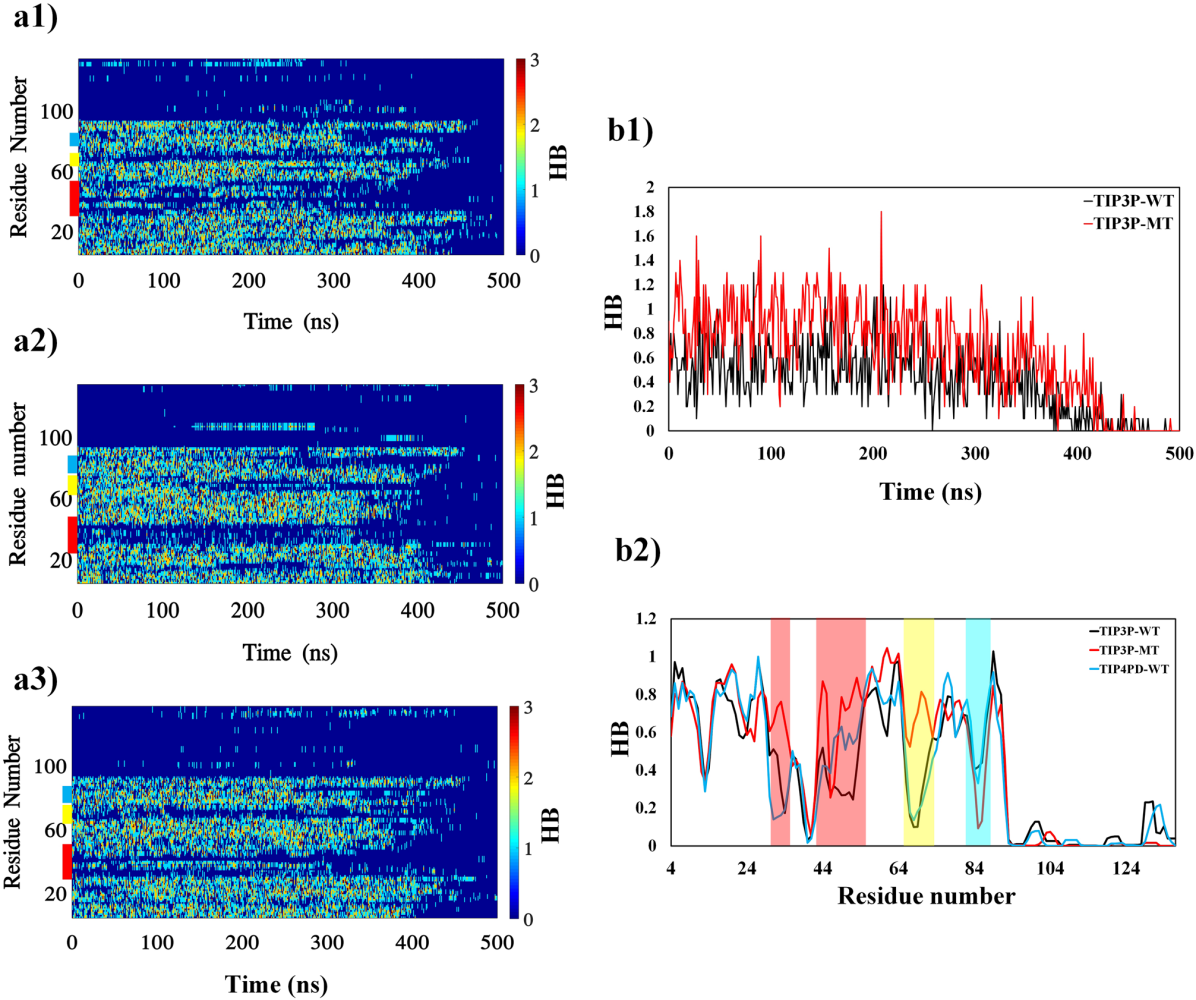
The internal hydrogen bond numbers ($HB$) along the TMD simulations. The figures in left panels show kymographs of three sets of the TMD simulations, protein wild type in TIP3P water model (TIP3P-WT), mutated protein in TIP3P water model (TIP3P-MT) and protein wild type in TIP4PD water model (TIP4PD-WT). Color bar in the right side indicates the values and the locations of the hot sites are colored as red, yellow and cyan in the left side of the plots indicating the first, second and third priorities, respectively. B1 compares the average $HB$ for valine and alanine residues in the hot sites (see text). B2 compares time averaged $HB$ of the residues for the three sets of the simulations*.*

To find the first opening regions of conformation transitions of αS, maps of structural features against the residual numbers in the TMD simulations. Figure 1a1 shows local deformations compared to the initial configuration of the protein in the simulations. Red color indicates high changes in conformations while stable parts are in dark blue. The change in local gyration radius ($\Delta {R}_{g}$) and the number of Hydrogen Bonds ($HB$) are also shown in Figs. 2a1 and 3a1, respectively. Figures 1, 2, and 3a1, all show similar patterns, indicating some regions that respond to deformations at initial stages. The end part of the protein (with residue numbers of after 90) having a very flexible coiled structure showed an irregular pattern, in contrast with structured parts of the protein. Figures 1, 2, and 3a1, several bands are shown in approximately the same positions as the thick colored lines on the left side of the figures showing most interchangeable regions along with structured parts of the protein. These band regions present the highest structural difference in every moment of the simulations. Longer bonds indicate the sites opened at early steps and formed the seeds for destroying the helical structure.

**Figure 4:**
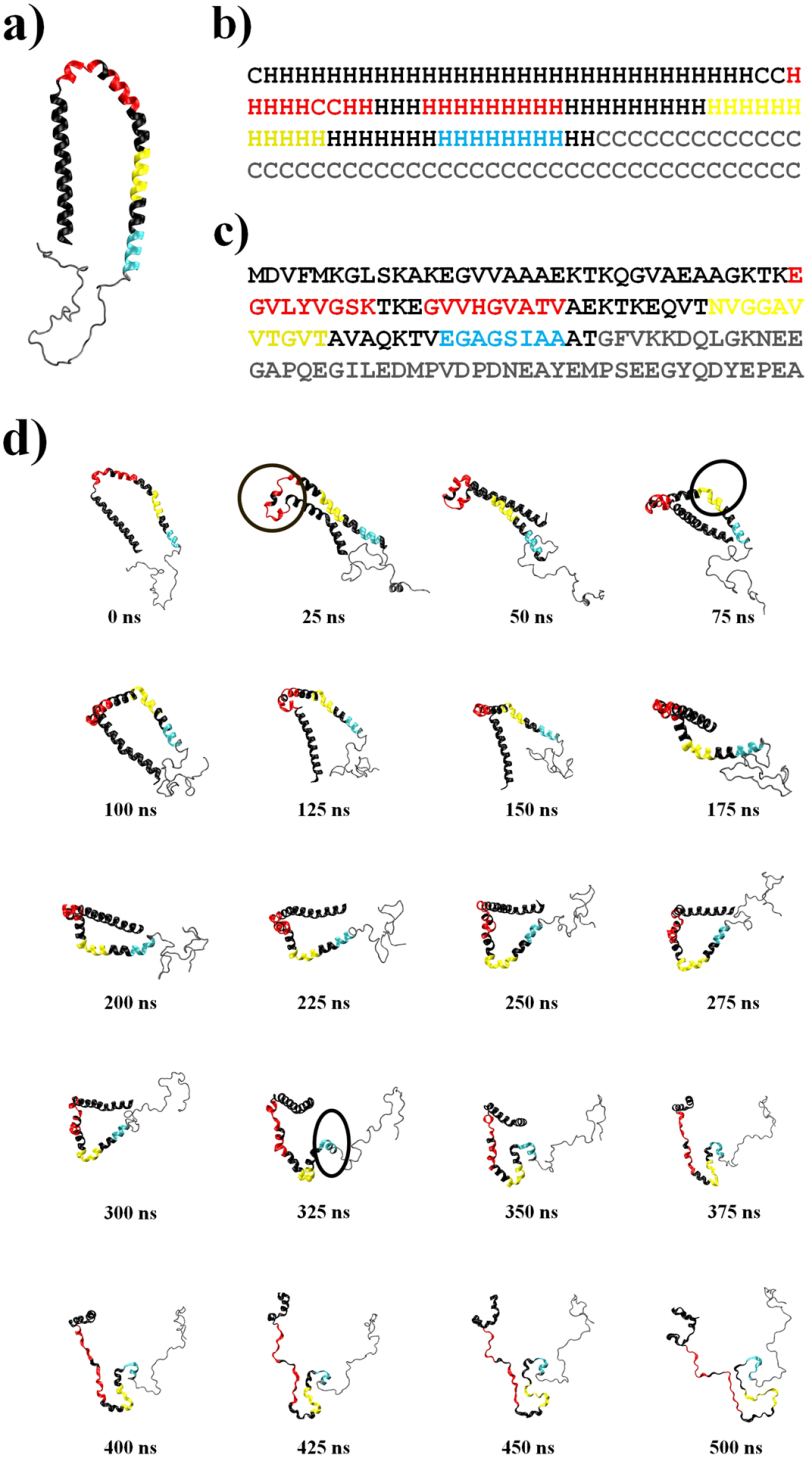
Hot regions in tertiary (**a**), secondary (**b**) and primary (**c**) structures of αS. Red, yellow and cyan colors are indicated the first, second and third priorities of hot regions, respectively. The gray color corresponds to the disorder part of αS. (**d**) Snapshots of αS intervals of 25 ns. The first, second and third priorities of hot sites are indicated red, yellow and cyan, respectively. The significant changes for each region are identified by circles in 25, 75 and 325 ns, respectively. The gray color corresponds to the disorder part of αS. Each configuration provided by VMD version 1.9.3^27^.

During the simulations, mentioned sites were opened in the following order: (i) the regions of (35–43) and (47–55), (ii) the regions of (65–75), and (iii) the region of (83–90) as highlighted by red, yellow, and cyan colors, respectively. The first opening regions (the hot regions) in tertiary, secondary and primary structures of αS are shown in Fig. 4a–c, respectively. Figure 4d shows the snapshots related to one of the TMD simulations in every 25 ns to better understand α–β conformational transition. There is a visible time priority of the opening regions (hot regions) in the protein dynamic along with the TMD simulation. The first opening sites are the regions in which significant structural changes occurred during αβT. The opening of these sites caused an increase in the local gyration radius and disappearance of the hydrogen bonds facilitating the transition of unstable conformations to the extended state. To investigate the thermodynamic stability of the results, all simulations were repeated for 300 K as reported in Fig. S1a1–a3 of the Supplementary Section. The RMSD, (${R}_{g}$), and $HB$ were compared with those in 310 K and the results have been presented in Fig. S1b1–b3 respectively. There is a small change of $RMSD$ in the hot regions while no significant changes were observed in (${R}_{g}$), and $HB$ plots. However, the position and priority of the hot regions are conserved in both temperatures of 300 K and 310 K, indicating the results are thermally stable and are expected to be visible in in-vitro experiments.

To ensure the irreversibility of the protein conformational transition, TMD simulations were repeated in reversed direction as reported in Supplementary Section. The RMSD, (${R}_{g}$), and $HB$ are obtained which show irregular patterns in the reversed direction of αβT, and explain the different pathways in the forward and backward directions (Fig. S2).

### Properties of hot sites

According to Anfinsen’s experiments, the amino acid sequence specifies the tertiary structure of proteins^28^. Propensities or conformational potentials were obtained from statistical analysis of secondary structure proteins, as the ratio of fractional occurrence of the residue in the given type of secondary structure to the fractional occurrence in all structures. According to the Chou–Fasman method^29^, the propensity of each residue in three types of secondary structures (Helix, extended, and coil) was calculated by Eq. (1).

1 $$P\left(R.S\right)=\frac{F\left(R.S\right)/F\left(R\right)}{Ns/N},$$

where $R$ and $S$ are amino acids and secondary structures; and ${N}_{S}$ and $N$ are total number of amino acids in conformation $S$ and the total number of amino acids in all secondary structures, respectively. Also, $F\left(R.S\right)$ indicates the number of occurrence of $R$ in $S$, and $P\left(R.S\right)$ is obtained as the propensity of R amino acid to be in $S$ structure.

Based on this principle, Chou and Fasman described three classes of the residue propensities in three types of protein secondary structures for the first time. The dataset used by Chou–Fasman for computing propensities of the amino acids was only limited to 15 proteins and 2,473 amino acids^29^. Over the years, the volume of datasets used to calculate the Chou–Fasmans̓ parameters has increased and finally the last applied dataset included a number of 2,164 proteins^30^. Since today, a number of nearly 150,000 proteins have been identified in the Protein Data Bank (PDB) database; and we updated the propensity of each amino acid in three types of secondary structures in the protein dataset consisting of more than 3,500 unique protein chains. Table 1 shows the propensity of each amino acid in the three types of secondary structures.

**Table 1:** The propensity of amino acids to be prefer in secondary structure types.

Amino acid	${P}_{\alpha }$	${P}_{\beta }$	${P}_{C}$
A	1.41	0.75	0.77
C	0.79	1.41	0.95
D	0.89	0.55	1.37
E	1.38	0.68	0.84
F	0.99	1.42	0.77
G	0.49	0.65	1.68
H	0.90	0.97	1.11
I	1.02	1.67	0.58
K	1.14	0.80	0.99
L	1.28	1.12	0.67
M	1.20	1.01	0.80
N	0.77	0.64	1.43
P	0.54	0.44	1.76
Q	1.25	0.79	0.89
R	1.19	0.88	0.89
S	0.80	0.89	1.25
T	0.76	1.25	1.08
V	0.85	1.87	0.62
W	1.06	1.34	0.74
Y	0.97	1.42	0.78

The amino acid names are shown in 1-letter characters. The ${P}_{\alpha }$, ${P}_{\beta }$ and ${P}_{C}$ are the propensity values of amino acids in helix, extended and coil structures, respectively.

#### Chameleonicity of hot sites

The chameleon site is defined as a distinct sequence that tends to be present in different secondary structure types of protein, meaning that these sites can adapted to different structures in response to their environment^31^. There are two major conditions for chameleon sequences; sequence propensity value to the beta structure should be more than 1 (${P}_{\beta }>1$; and secondary structure of protein should be in helix or coil conformations^32^. Therefore, αS helical regions that tend to have more than one beta value are good candidate of these regions.

**Figure 5:**
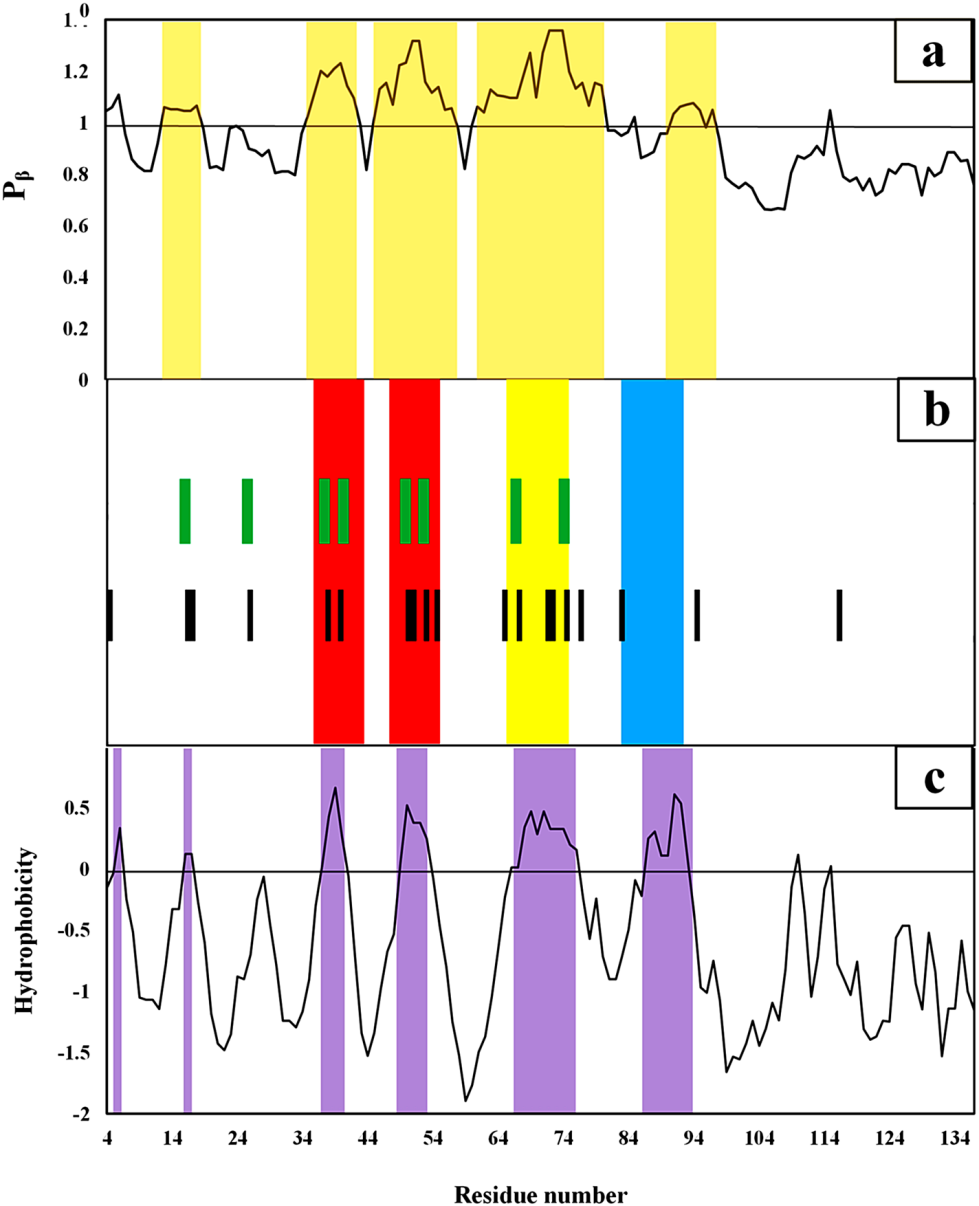
Sequence-dependent properties of hot sites over the sliding window. (**a**) Shows the propensity values of the residues to the extended structures. The light brown color bands correspond to the chameleonic regions. The hot regions of αS are shown in (**b**) panel. The black lines show the location of valine residues while the green lines are the G–V sites, respectively. (**c**) Shows the hydrophobicity values of αS residues. The purple bands are related to the high hydrophobic sites along αS.

To identify chameleonic sites, propensities to extended conformations were averaged over the sliding windows (Fig. 5a). The regions of (14–18), (35–42), (46–57), (61–80), and (90–94) were identified as chameleon sites with a high tendency for beta structure. These regions are the most likely to form the β-strands in αβT. A comparison of the hot regions (Fig. 5b) indicates the chameleonicity of these regions which help the protein to lose the helical configuration at the onest of the protein αβT. These sites are rich in valine and glycine residues, which together form a specific pattern called the G–V pattern (Fig. 5b). The G–V pattern gives high flexibility to hot regions that play key roles in conformational transition, which as described in “Role of G–V pattern in hot sites”.

#### Hydrophobicity of hot sites

Alternating polar and nonpolar residues create alternating hydrophobic and hydrophilic faces in the protein and facilitate beta-strand formation^33^. There are different alternating polar and nonpolar ($N/P$) patterns in aggregation-prone proteins^34^, which diversities in $N/P$ pattern causing specific beta-sheet forms under different conditions^35,36^. The αS sequence has alternating polar and nonpolar sites stimulate the protein to form aggregates and nonpolar sites usually have high hydrophobicity that results in the tendency to formation of amyloid fibrils^37^. According to the Roseman’s hydropathy scales^38^, Fig. 5c shows hydrophobicity values averaged over the sliding windows. The regions of (6–7), (16–17), (37–40), (49–53), (66–76), and (87–93) with values over 0 were identified as hydrophobic sites of αS. These hydrophobic regions are at the heart of hot sites and make them more potent for the formation of amyloid fibrils. In fact, these middle hydrophobic cores can act as a driving force for β-strand formation^39^ leading to the initiation of αβT from these sites and subsequent sites. Figure S3 shows the relationship between hydrophobicity and propensity values. The values of 0.61 and − 0.37 were obtained from the correlation between hydrophobicity and ${P}_{\beta }$ and ${P}_{\alpha }$, respectively. This means that, hydrophobic sites tend to lose helical structures and convert them to extended structures. Although, both reported correlation values are not very high but they are significant at p-value of $<0.001$*.*

### Role of G–V pattern in hot sites

The valine is an aliphatic and hydrophobic amino acid with the highest propensity to the beta structure in comparison with the other amino acids^30^. As the valine is small and has a non-reactive side chain, the valine-rich regions are less restricted in conformational changes of the protein^40,41^. As it is more difficult to adopt valine-rich regions (hot regions) with the regular α-helical conformation these regions prefer to be in beta-sheet states. Absence of ring in the valine side-chain creates the main-chain amid hydrogens (NH), which have not been protected against solvent hydrogens^40^ resulting in the smallest environmental changes around these regions that make them to convert the helical state to an extended one. Therefore, the presence of valine in hot regions provides an intrinsic tendency to lose helical structure and make these regions highly susceptible to long-term conformational transition. It is noteworthy that, the glycine is placed next to the valine residues in the valine-rich sites. Figure 5b indicates location of valine residues (black bars) and V–G pairs (green bars) over the protein sequence. There is a concentration of black and green lines in the hot regions which indicates the role of G–V pattern in the formation of these sensitive sites. The G–V sites are ending part of repetitive sequence of KTKEGV known to be able to form helical structure upon binding of the protein to the mitochondrial membrane^42^. It appears that the presence of the G–V sites acts as a key part of initiating conformational transition from helical to extended structures. The presence of glycine next to the valine results from intrinsic behavior of glycine in the $\varphi /\psi$ space.

Intrinsic behavior of amino acids plays a major role in their conformational preferences in the $\varphi /\psi$ space^43,44^ creating a set of dihedral angles to special values that form secondary structure types^45^ and is identified in special regions of the Ramachandran plot^46^. Intrinsic behavior of glycine allows its $\varphi$ and $\psi$ angle values to fall in wide range^47^ and its presence next to the valine causes it to act as hinge donating that stimulates the G–V regions during the αβT.

**Figure 6:**
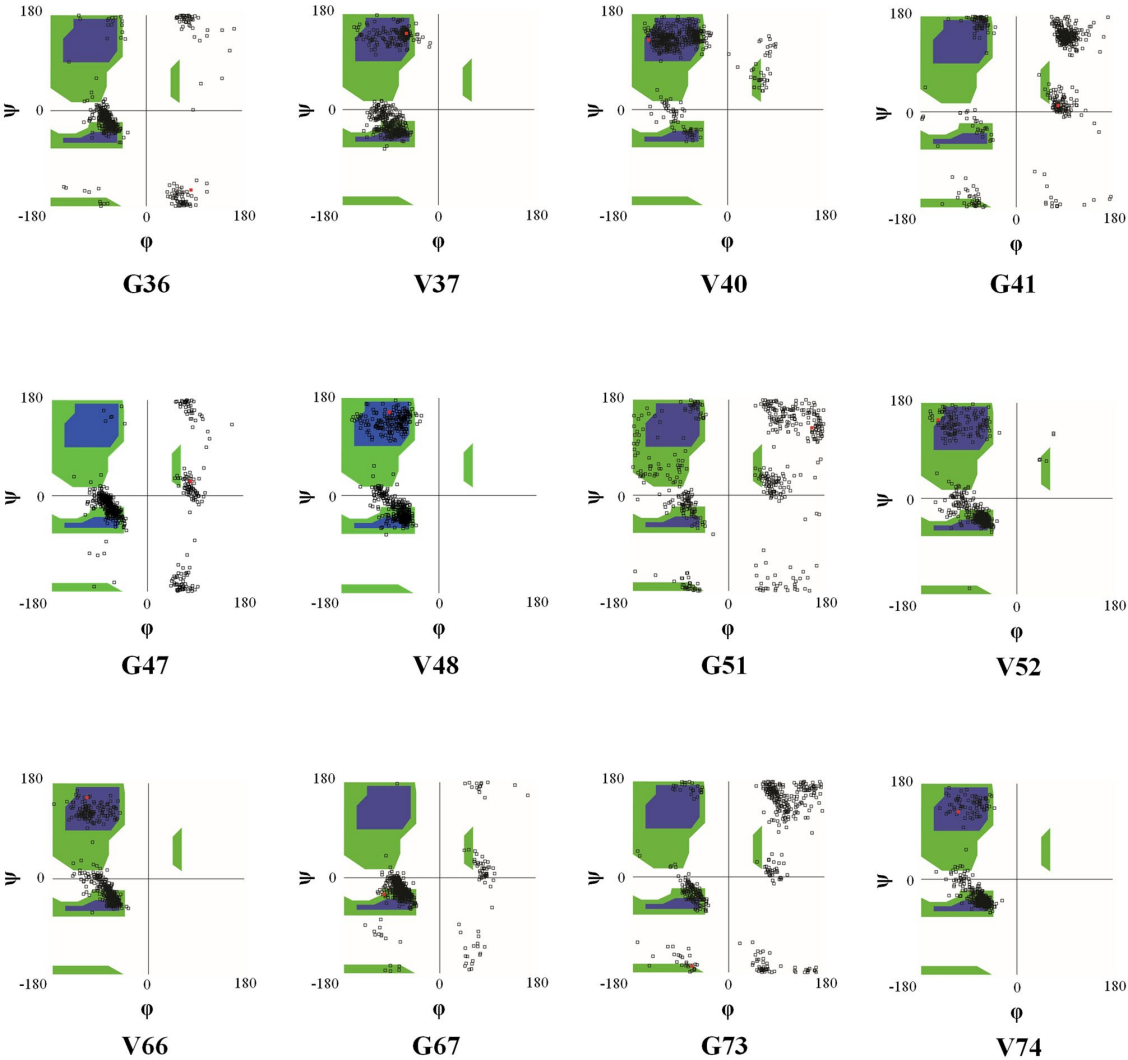
The distributions of dihedral angle values of G–V sites during the simulation time in the $\varphi /\psi$ space. The white regions are sterically disallowed for all amino acids except glycine. The blue regions correspond to the allowed regions namely the helix and extended conformations. The green areas show outer limit regions. The black points indicate the distribution of dihedral angle values of each residue during the simulation time.

All the G–V regions of hot sites were identified and their behavior was investigated in the $\varphi /\psi$ space. The pictures in Fig. 6 represent dihedral angle values of G–V regions in hot sites during the TMD simulations. Distributions of valine dihedral angle values were in helix and beta areas of the plot, while the glycine ones started from the helix region and continued in the other parts of Ramachandran plot during the TMD simulation. This means that the presence of the glycine alongside the valine residue gives a great deal of flexibility in hot sites and made conformational transition from helical to extended states more convenient than the other parts of protein.

**Figure 7:**
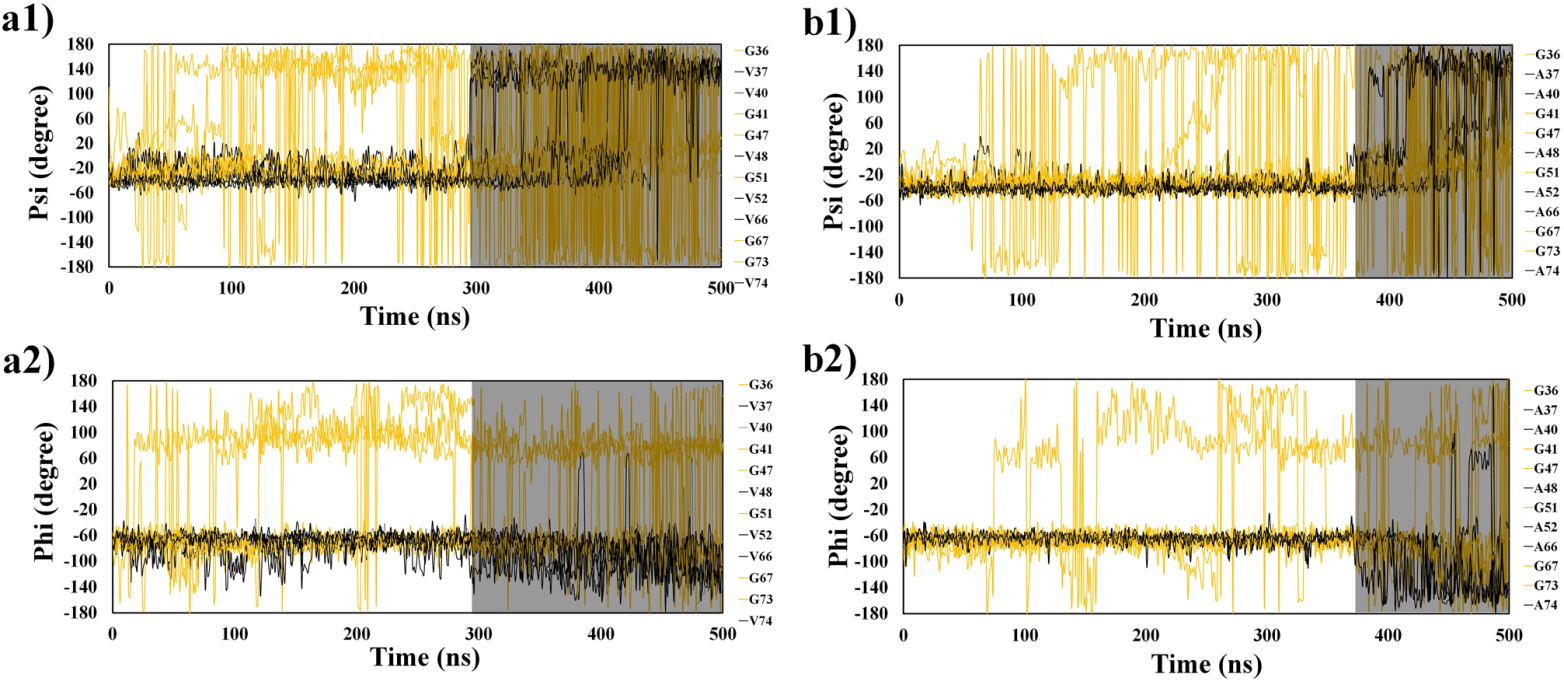
Dihedral angle ($\psi$ and $\varphi$) values of G–V sites for wild (**a1**, **a2**) and mutated (**b1**, **b2**) proteins during the simulations. The black and orange lines are dihedral angle values of valine and glycine, respectively. The shadowed part in each plot indicate the transition of from helix to extended conformations.

As shown in Fig. 7a1 and a2, the $\psi$ and $\varphi$ angle values indicate a two-state structure for valine while the glycine ones are more fluctuating. To ensure the valine effect on the αβT, the valine residues of the G-V regions were mutated to the Alanine residues and TMD was performed on mutated protein.

#### The alanine scanning of G–V sites

The alanine scanning^48^ is a useful technique used to determine the contribution of valine residue to the guidance of helical structures towards extended structures. Since the alanine is a non-bulky and chemical inert amino acid^30^ with highest propensity to the helix structure (See Table 1), it was selected as the best candidate instead of valine. To understand role of the valine in hot regions, all the valine residues in the G–V sites were mutated to the alanine and TMD simulation was performed on the αS mutated structure. Plots for the $RMSD$, (${R}_{g}$), and $HB$ were obtained and the results have been presented in Figs. 1, 2, and 3a2. As can be seen, valine mutation to the alanine residue decreased flexibility of hot sites and reduced color pattern of hot bands showing the αβT is less favorable compared to the wild type. The tendency of the protein to keep the helical structure reduced fluctuation of dihedral angles and αβT occurred much later for mutant protein (shadow regions in Fig. 7b1, b2). Therefore, the intrinsic propensity of each amino acid in a variety of secondary structures has a significant effect on conformational transition of the protein.

More precisely, the mean $RMSD$, (${R}_{g}$), and $HB$ of valine and alanine residues of the hot regions compared between the wild and mutant variants (Figs. 1, 2, 3b1). The $RMSD$ values of mutant protein are smaller than in the wild type, indicating the mutate protein retains the helical structure more than the wild type. The difference of (${\Delta R}_{g}$) values between the wild and mutant types after 350 ns shows the tendency of the alanine residues to be in helical structure. Also, smaller values of wild type $HB$s indicate that the helical state in protein clears the protein faster than the mutant form. The mean time of the variables for each residues has also been plotted to compare the behavior of the protein (Figs. 1, 2, 3b2). In general, the mutate protein exhibits different behaviors across the $RMSD$, (${R}_{g}$), and $HB$ curves (Specially the hot regions) compare to the wild type.

### Influence of TIP4PD water on protein αβT

To investigate the effect of the water model on the protein conformational transition, TMD simulations are repeated with TIP4PD water model for the protein wild type. The TIP4PD water model reproduced the most accurate conformational ensemble for intrinsically disordered proteins which is recommended for simulating of disordered proteins^49^. The $RMSD$, (${R}_{g}$), and $HB$ plots were obtained and the results have been presented in Figs. 1, 2, and 3a3 respectively. Although, the TIP4P-D water model did not affect the location of hot regions but a moderate change was observed in the color pattern in Figs. 1, 2, and 3a3 compared to the simulations in TIP3P water model. For a better comparison, the Figs. 1, 2, and 3b2, show the average values along the simulations. The smaller values of $RMSD$, (${R}_{g}$), and $HB$ in the hot regions indicate that the TIP4PD water model helps to preserve more the helical structure of these regions along the protein conformational transition and the TIP4PD water model has delayed the opening of the second region more than the other hot sites. However, the position and priority of the hot regions are conserved and the conclusion of the paper do not change. The low sensitivity of the hot regions indicates that the amino acid propensity to the secondary structure types plays a more dominant role than the water model in the conformational transition which shows TIP4PD water model effects on αβT process speed.

### Conformational clusters in αβT pathway

**Figure 8:**
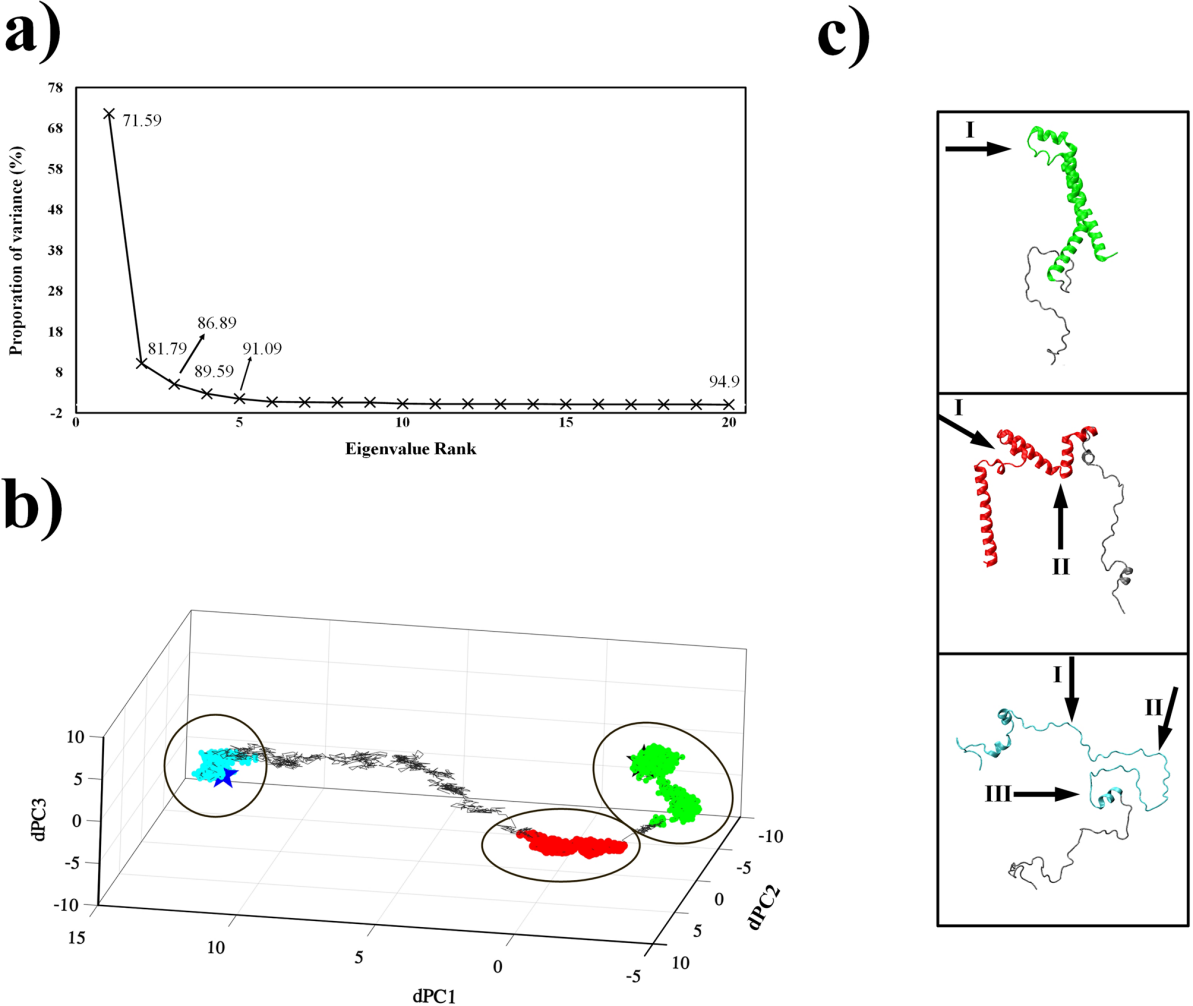
Principal components analysis. (**a**) The contributions of the eigenvalues of $dPC$s for the variance. (**b**) Presentation of the structures in the space defined by the first three $dPC$s. The conformational clusters from the first to third obtained by peak-picking method are colored by green, red and cyan, respectively. The black and dark blue stars indicate the helical (initial) and extended (target) structures, respectively. A centroid member of each clusters are shown in (**c**) the black arrows point to the active regions in the representative configurations. The gray line shows the trajectory (αβT).

The $dPCA$ was applied to the TMD trajectories to characterize significant conformers during αβT. As shown in Fig. 8a, the eigenvalue contribution of $dPC$ indicates that the first $dPC$ is accounted for more than 70% of the overall variance and over 85% of motions are covered in the first three components. Therefore, the $dPC$ space is defined by the first three $dPC$ s and conformations are clustered into three clusters. The clustering was performed using the peak-picking algorithm to identify isolated peaks distributed in principal components of the configurations along the TMD trajectories, corresponding to discrete clusters^50^. According to Fig. 8b, three clusters with the population of 18.34%, 47.20% and 13.40% are respectively shown in green, red, and cyan colors in $dPC$ space. Green and cyan clusters contain the configurations that are respectively close to helical (initial) and extended (target) states. A centroid point of each group was selected as representative conformation and their RMSD compared to initial and target configurations are reported in Table 2.

**Table 2:** The pairwise $RMSD$ between helical and extended conformations with each of the clusters representatives.

Representative structure	Helical structure	Extended structure
Helical	0	24.22
Cluster 1	9.15	21.14
Cluster 2	17.19	18.98
Cluster 3	20.28	6.51
Extended	24.22	0

An accurate view of the RMSD values indicated the structural similarity between representatives. The representative of the second cluster could be between the helical and extended states. This cluster with 47.20% of total population showing the most configurations during protein conformational transition. These configurations are related to the early stages in αβT and first and second priorities of hot sites are active in them. While the configurations of the first and third clusters are more similar to the helical and extended states, respectively. It can also be seen that the hot regions with the highest priority are active in the first cluster configurations while all the hot sites are involved in the third cluster configurations (see Fig. 8c).

## Conclusions

In this study, a comparison was found for monomeric αβT of αS in different conditions using TMD simulations. In the transition pathway, critical sites with a key role in the amyloid fibril formation were identified in three priorities (i) (35–43) and (47–55), (ii) (65–75), and (iii) (83–90), respectively. The regions with good overlap, as well as with aggregation-prone regions have been introduced by Pawar et al.^51^. All these sites are highly hydrophobic and tend to form extended conformation. Chameleonic properties were observed for these regions. In critical regions, the presence of G–V patterns donated high flexibility to facilitate the conformational transition between helical and extended states. Previous studies^42,52^ also showed that, 5 missense mutations, namely A30P, E46K, H50Q, G51D, and A53T increase the fibrillation rate in the first priority region.

Experimental findings showed that the peptides designed on the central hydrophobic region (61–82 residues) have high efficiency in blocking of αS aggregation and fibrillation^51,53^. In another study conducted by our group, it has been shown that therapeutic peptides designed on the regions of (46–53) and (70–75) were able to block αS aggregation and fibrillation and open toxic oligomers, respectively^53^. Our new findings suggest that, in addition to the two studied regions, the region of (35–43) is a good candidate for designing efficient therapeutic peptides.

The increasing in the gyration radius and the decreasing the number of hydrogen bond in hot regions resulted in the formation of unstable and temporary conformations. The results indicated that G–V patterns play a major role in the high flexibility of the hot sites in protein conformational transition and the mutation of the valines to the alanine residues increased the tendency of the protein to keep helical structure. The TIP4PD water model does not affect the position and priority of hot regions and just delays their conformational transitions specially in the second region. The trajectory can be categorized into three structural clusters along the αβT. The representative of each cluster was compared to the helical and extended structures through RMSD calculations and their active hot regions were observed. The results of this study highlight the mechanism of αβT in the αS and may be useful in designing a better generation of amyloidogenic peptides.

## Methods

### Selecting of starting and ending points in α–β transition

TMD simulations are needed to select two stable structures for starting and ending points. The helical state is structured as well as its full-length PDB file is available in the Protein Data Bank (determined using nuclear magnetic resonance (NMR) spectroscopy, pdb ID: 2KKW^54^). The structure of αS fibrils at atomic resolution (pdb ID: 2N0A^55^) was selected for the ending point of TMD simulations. This structure was determined using nuclear magnetic resonance (NMR) spectroscopy containing full-length protein chains in extended states. This selection represents a single chain that interacts with other amyloid fibrils chains. This helps TMD simulations to sample the helical transition to amyloid fibril structure. Since we are locally focused on only one chain of amyloid fibrils, polymorphic properties do not affect the results (See Supplementary Fig. S4).

### TMD procedure

The αS structures in both helical and extended conformations were obtained from the Protein Data Bank (PDB) database. The first model of NMR structure with PDB ID of 2KKW and the first chain of NMR structure with PDB ID of 2N0A were selected as helical (folded) and extended (amyloid) structures, respectively.

In this study, 3 TMD^26^ simulations were performed to focus on the monomeric conformational transition of αS. The protein alpha–beta transition and reversed transition were investigated to find critical regions in the structural transitions and for understanding the conditions to create β-forming regions. In the αβT simulations, helical structure (based on PDB ID of 2KKW) was considered as initial conformation forced toward extended configuration, and in reversed transition, extended structure (based on PDB ID of 2N0A) was considered as initial structure.

Moreover, another set of simulations was performed on the αS mutated structure to prove that valine plays a key role in protein conformational transition. All the valine residues at the hot sites (see section “The alanine scanning of G–V sites”) of αS helical structure (based on PDB ID of 2KKW) mutated to the alanine were modeled by MODELLER 9.20^56^ and energy was minimized in constructed 3D model.

TMD was performed in cubic box of 9,086 water molecules which was neutralized by the addition of 9 Na^+^ ions. TIP3P^57^ was used to model water molecules and CHARMM27 force-field parameters were applied. In all simulations, 50,000 minimization steps of the conjugated gradient were done for frozen protein Cα atoms with a positional harmonic force of 10 kcal mol^−1^ Å^2^ and heating up to 310 K over 300 ps for NPT ensemble. The Langevin thermostat^58^ and the Nose–Hoover barostat^59^ were applied to keep temperature and pressure at 310 K and 1.01325 bar, respectively. A short-range cutoff of 10 Å was treated for non-bonded interactions, and long-range electrostatic interactions were considered using the Particle Mesh Ewald (PME) method^60^ combined with periodic boundary conditions. At initial stages of equilibration, a time step of 1 fs was done while, the SETTLE algorithm^61^ was used to keep hydrogen-heavy atom bond lengths frozen during simulation at subsequent steps thus, a time period 2 fs was adopted. After equilibration, all the restraints were gradually removed and TMD was carried out for 500 ns. During the TMD simulations, our NPT ensemble included an additional energy term based on the RMSD of protein residues that force the molecule concering a prescribed target structure. The time-dependent energy function was as follows.

2 $${U}_{TMD}=\frac{1}{2}NK{\left(R-\rho \left(t\right)\right)}^{2}$$

where, $N$ represents the number of ${C}_{\alpha }$ atoms in protein backbone, $K$ is harmonic force constant set as 200 kcal mol^−1^ Å^−2^, and $R$ is the Root Mean Square Deviation (RMSD) between a conformation at time $t$ and target conformation. $\rho \left(t\right)$ is reference RMSD value at time $t$ that linearly decreased from 24.22 to 0 Å within the TMD simulation time. The TMD forces were applied to the alpha carbons during the simulation. The center of mass and protein orientation was fixed during the simulation to prevent molecular rotation. The NAMD 2.13 program^62^ was utilized for the TMD simulations and all related analyses were done using the VMD 1.9.3^27^.

To investigate the effect of water model on protein conformational transition, similar TMD simulations are performed using the TIP4PD water model^49^. Since the updated version of CHARMM force field by Mackrell published in 2019, July (https://mackerell.umaryland.edu/charmm_ff.shtml) didn’t have the parameters and topology files for TIP4PD water model, we applied the TIP4P-2005 files and modified the charge, energy ($\varepsilon$) and the minimum distance $({R}_{min})$ of the water atoms according to the parameters reported in the David Shaw’s paper^49^. A small TIP4PD water box (100 Å per dimension) was fabricated using the PACKMOL package^63^ and relaxed with a 2 ns regular MD simulation. This obtained box is applied to make the solvent box for the system. The previous standard TMD simulations protocol was performed. TMD simulations performance decreased by %10 due to the use of the TIP4PD water model.

### Applying the sliding window to fragmental analysis

To consider the effect of neighbor residues, all the properties were statistically averaged over a sliding window of residues along the protein chain. The average value of every property was assigned to the middle residues of each sliding window in the αS sequence. The size of the sliding window can be between 3 and 10 residues^64^ but since, in biological concentration, the probability of amyloid formation is low for small protein fragments, larger sizes are more suitable for window selection^65^. In this paper, number of 7 was selected as the size of the sliding window (See Supplementary Fig. S5). So, every feature was computed using the sliding window, and has been issued for the 4th to 137th residues of αS. The sliding window was used for fragmental averaging of the protein propensity, hydrophobicity, gyration radius, and the number of hydrogen bonds.

### Analysis of TMD trajectories

To understand conformational changes during the protein αβT, several types of analyses were performed on the TMD trajectories. The radius of gyration (${R}_{g}$) and RMSD values of the alpha carbons, Hydrogen bonds (the bonds with a bond length cutoff of 3.0 Å and an angle cutoff of 20°), and dihedral angles of key residues of critical sites for each frame of TMD trajectory were calculated over sliding windows using VMD^27^.

Principal Component Analysis of backbone dihedral angles $(dPCA$) of the TMD trajectory was performed in CARMA version 1.7^66^. The clustering method was performed based on a peak-picking algorithm^67^ embedded in the CARMA applied to three-dimensional distributions of principal components derived from the TMD trajectory. The first three principle components (three largest $dPC$s) were considered to identify prominent molecular configurations for populated clusters in three-dimensional $dPC$ space.

## Supplementary information

Supplementary information.

## References

[1] Hardy J, Selkoe DJ (2002). The amyloid hypothesis of Alzheimer's disease: progress and problems on the road to therapeutics. Science.

[2] Singleton A (2003). α-Synuclein locus triplication causes Parkinson's disease. Science.

[3] Anguiano M, Nowak RJ, Lansbury PT (2002). Protofibrillar islet amyloid polypeptide permeabilizes synthetic vesicles by a pore-like mechanism that may be relevant to type II diabetes. Biochemistry.

[4] Dehay B (2015). Targeting α-synuclein for treatment of Parkinson's disease: mechanistic and therapeutic considerations. Lancet Neurol..

[5] Polymeropoulos MH (1997). Mutation in the α-synuclein gene identified in families with Parkinson's disease. Science.

[6] Goedert M (2001). Alpha-synuclein and neurodegenerative diseases. Nat. Rev. Neurosci..

[7] Prots I (2018). α-Synuclein oligomers induce early axonal dysfunction in human iPSC-based models of synucleinopathies. Proc. Natl. Acad. Sci. USA.

[8] Lee J-E (2018). Mapping surface hydrophobicity of α-synuclein oligomers at the nanoscale. Nano Lett..

[9] Lashuel HA, Overk CR, Oueslati A, Masliah E (2013). The many faces of α-synuclein: from structure and toxicity to therapeutic target. Nat. Rev. Neurosci..

[10] Hajipour MJ (2017). Advances in Alzheimer’s diagnosis and therapy: The implications of nanotechnology. Trends Biotechnol..

[11] Arosio P (2016). Kinetic analysis reveals the diversity of microscopic mechanisms through which molecular chaperones suppress amyloid formation. Nat. Commun..

[12] Poewe W (2017). Parkinson disease. Nat. Rev. Dis. Primers.

[13] Sahoo A, Matysiak S (2019). Computational insights into lipid assisted peptide misfolding and aggregation in neurodegeneration. Phys. Chem. Chem. Physics.

[14] Wang C (2016). Versatile structures of α-synuclein. Front. Mol. Neurosci..

[15] Theillet F-X (2016). Structural disorder of monomeric α-synuclein persists in mammalian cells. Nature.

[16] Breydo L, Wu JW, Uversky VN (2012). α-Synuclein misfolding and Parkinson's disease. Biochim. Biophys. Acta.

[17] Bartels T, Choi JG, Selkoe DJ (2011). α-Synuclein occurs physiologically as a helically folded tetramer that resists aggregation. Nature.

[18] Burré J (2013). Properties of native brain α-synuclein. Nature.

[19] Cookson MR (2009). α-Synuclein and neuronal cell death. Mol. Neurodegener..

[20] Meade RM, Fairlie DP, Mason JM (2019). Alpha-synuclein structure and Parkinson’s disease–lessons and emerging principles. Mol. Neurodegener..

[21] Pacheco C, Aguayo LG, Opazo C (2012). An extracellular mechanism that can explain the neurotoxic effects of α-synuclein aggregates in the brain. Front. Physiol..

[22] Wei G (2017). Self-assembling peptide and protein amyloids: from structure to tailored function in nanotechnology. Chem. Soc. Rev..

[23] Ludtmann MH (2018). α-synuclein oligomers interact with ATP synthase and open the permeability transition pore in Parkinson’s disease. Nat. Commun..

[24] Fusco G (2017). Structural basis of membrane disruption and cellular toxicity by α-synuclein oligomers. Science.

[25] Qin Z, Buehler MJ (2010). Molecular dynamics simulation of the α-helix to β-sheet transition in coiled protein filaments: evidence for a critical filament length scale. Phys. Rev. Lett..

[26] Schlitter J, Engels M, Krüger P, Jacoby E, Wollmer A (1993). Targeted molecular dynamics simulation of conformational change-application to the T↔ R transition in insulin. Mol. Simul..

[27] Humphrey W, Dalke A, Schulten K (1996). VMD: visual molecular dynamics. J. Mol. Graph..

[28] Anfinsen CB (1973). Principles that govern the folding of protein chains. Science.

[29] Chou PY, Fasman GD (1974). Prediction of protein conformation. Biochemistry.

[30] Costantini S, Colonna G, Facchiano AM (2006). Amino acid propensities for secondary structures are influenced by the protein structural class. Biochem. Biophys. Res. Commun..

[31] Kabsch W, Sander C (1984). On the use of sequence homologies to predict protein structure: identical pentapeptides can have completely different conformations. Proc. Natl. Acad. Sci..

[32] Gendoo DM, Harrison PM (2011). Discordant and chameleon sequences: their distribution and implications for amyloidogenicity. Protein Sci..

[33] Zhang S, Corin K (2018). Peptide Applications in Biomedicine, Biotechnology and Bioengineering.

[34] Wang W, Hecht MH (2002). Rationally designed mutations convert de novo amyloid-like fibrils into monomeric β-sheet proteins. Proc. Natl. Acad. Sci..

[35] Yu T-G, Kim H-S, Choi Y (2019). B-SIDER: computational algorithm for the design of complementary β-sheet sequences. BioRxiv.

[36] Olajos G (2018). Peripheral cyclic β-amino acids balance the stability and edge-protection of β-sandwiches. Org. Biomol. Chem..

[37] Gasteiger E (2005). The Proteomics Protocols Handbook.

[38] Roseman MA (1988). Hydrophilicity of polar amino acid side-chains is markedly reduced by flanking peptide bonds. J. Mol. Biol..

[39] Waxman EA, Mazzulli JR, Giasson BI (2009). Characterization of hydrophobic residue requirements for α-synuclein fibrillization. Biochemistry.

[40] Larsson SC, Markus HS (2017). Branched-chain amino acids and Alzheimer’s disease: a Mendelian randomization analysis. Sci. Rep..

[41] Kathuria SV, Chan YH, Nobrega RP, Özen A, Matthews CR (2016). Clusters of isoleucine, leucine, and valine side chains define cores of stability in high-energy states of globular proteins: sequence determinants of structure and stability. Protein Sci..

[42] Ferreira M, Massano J (2017). An updated review of Parkinson's disease genetics and clinicopathological correlations. Acta Neurol. Scand..

[43] Childers MC, Towse C-L, Daggett V (2016). The effect of chirality and steric hindrance on intrinsic backbone conformational propensities: tools for protein design. Protein Eng. Des. Sel..

[44] Schweitzer-Stenner R, Toal SE (2014). Entropy reduction in unfolded peptides (and proteins) due to conformational preferences of amino acid residues. Phys. Chem. Chem. Phys..

[45] Jiang F, Han W, Wu Y-D (2013). The intrinsic conformational features of amino acids from a protein coil library and their applications in force field development. Phys. Chem. Chem. Phys..

[46] Ramachandran GN (1963). Stereochemistry of polypeptide chain configurations. J. Mol. Biol..

[47] Maxwell PI, Popelier PL (2017). Unfavorable regions in the ramachandran plot: Is it really steric hindrance? The interacting quantum atoms perspective. J. Comput. Chem..

[48] Morrison KL, Weiss GA (2001). Combinatorial alanine-scanning. Curr. Opin. Chem. Biol..

[49] Piana S, Donchev AG, Robustelli P, Shaw DE (2015). Water dispersion interactions strongly influence simulated structural properties of disordered protein states. J. Phys. Chem. B.

[50] Sivanesam K, Andersen N (2019). Pre-structured hydrophobic peptide β-strands: a universal amyloid trap?. Arch. Biochem. Biophys..

[51] Pawar AP (2005). Prediction of “aggregation-prone” and “aggregation-susceptible” regions in proteins associated with neurodegenerative diseases. J. Mol. Biol..

[52] Selvaraj S, Piramanayagam S (2019). Impact of gene mutation in the development of Parkinson's disease. Genes.

[53] Rezaeian N, Shirvanizadeh N, Mohammadi S, Nikkhah M, Arab SS (2017). The inhibitory effects of biomimetically designed peptides on α-synuclein aggregation. Arch. Biochem..

[54] Rao JN, Jao CC, Hegde BG, Langen R, Ulmer TS (2010). A combinatorial NMR and EPR approach for evaluating the structural ensemble of partially folded proteins. J. Am. Chem. Soc..

[55] Tuttle MD (2016). Solid-state NMR structure of a pathogenic fibril of full-length human α-synuclein. Nat. Struct. Mol. Biol..

[56] Šali A, Blundell TL (1993). Comparative protein modelling by satisfaction of spatial restraints. J. Mol. Biol..

[57] Jorgensen WL, Chandrasekhar J, Madura JD, Impey RW, Klein ML (1983). Comparison of simple potential functions for simulating liquid water. J. Chem. Phys..

[58] Adelman S, Doll J (1976). Generalized Langevin equation approach for atom/solid-surface scattering: general formulation for classical scattering off harmonic solids. J. Chem. Phys..

[59] Martyna GJ, Tobias DJ, Klein ML (1994). Constant pressure molecular dynamics algorithms. J. Chem. Phys..

[60] Petersen HG (1995). Accuracy and efficiency of the particle mesh Ewald method. J. Chem. Phys..

[61] Miyamoto S, Kollman PA (1992). Settle: an analytical version of the SHAKE and RATTLE algorithm for rigid water models. J. Comput. Chem..

[62] Phillips JC (2005). Scalable molecular dynamics with NAMD. J. Comput. Chem..

[63] Martínez L, Andrade R, Birgin EG, Martínez JM (2009). PACKMOL: a package for building initial configurations for molecular dynamics simulations. J. Comput. Chem..

[64] Tian J, Wu N, Guo J, Fan Y (2009). Prediction of amyloid fibril-forming segments based on a support vector machine. BMC Bioinform..

[65] Ahmed AB, Znassi N, Château M-T, Kajava AV (2015). A structure-based approach to predict predisposition to amyloidosis. Alzheimer.

[66] Glykos NM (2006). Software news and updates carma: a molecular dynamics analysis program. J. Comput. Chem..

[67] Baltzis, A. S., Koukos, P. I. & Glykos, N. M. Clustering of molecular dynamics trajectories via peak-picking in multidimensional PCA-derived distributions. *arXiv preprint arXiv:.04024* (2015).

