# Supplementary material for: The hot sites of α-synuclein in amyloid fibril formation
Source: Sci Rep. 2020 Jul 22;10:12175. doi: 10.1038/s41598-020-68887-2 (PMC7376172; doi:10.1038/s41598-020-68887-2)
Supplement: Supplementary file 1 — Supplementary information. [file 41598_2020_68887_MOESM1_ESM.docx]

**The Hot Sites of α-Synuclein in Amyloid Fibril Formation**

**Anahita Khammari^1^, Seyed Shahriar Arab^1,*^ and Mohammad Reza Ejtehadi^2,3,*^**

^1^Department of Biophysics, School of Biological Sciences, Tarbiat Modares University, Tehran, Iran

^2^Physics Department, Sharif University of Technology, P.O. Box 11155-9161, Tehran, Iran

^3^School of Nano-Science, Institute for Research in Fundamental Sciences (IPM), Tehran, Iran

^*^[sh.arab@modares.ac.ir](mailto:sh.arab@modares.ac.ir)

^*^[ejtehadi@sharif.edu](mailto:ejtehadi@sharif.edu)

^*^Correspondences should be sent to these authors


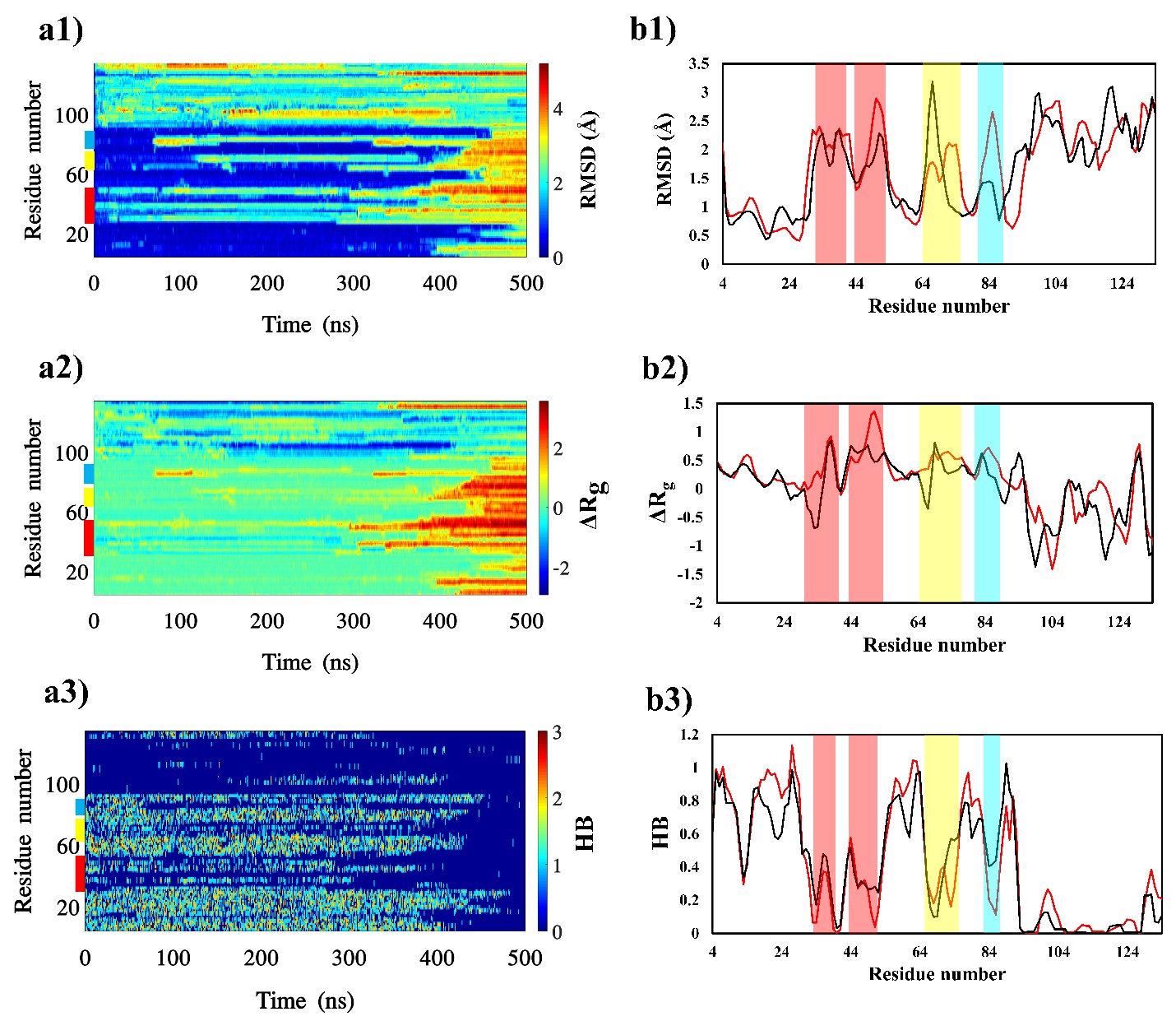


**Fig S1:** Structural properties during the TMD simulation for protein *in 300K*. a1) The $RMSD$ of alpha carbons from the initial configuration. a2) The difference between gyration radius ($\Delta R_{g}$) of of each frame configurations with the first frame during TMD trajectories. a3) The internal hydrogen bond numbers ($HB$) along the time simulations. All the properties are calculated over the sliding windows in compare to initial configurations as the reference point. The locations of the hot sites are colored as red, yellow and cyan in the left side of the plots. b1, b2 and b3 compares mean time RMSD, $\Delta R_{g}$ and $HB$ of the residues along the TMD simulations. The black and red curves are shown the simulations in 310 and 300K, respectively.


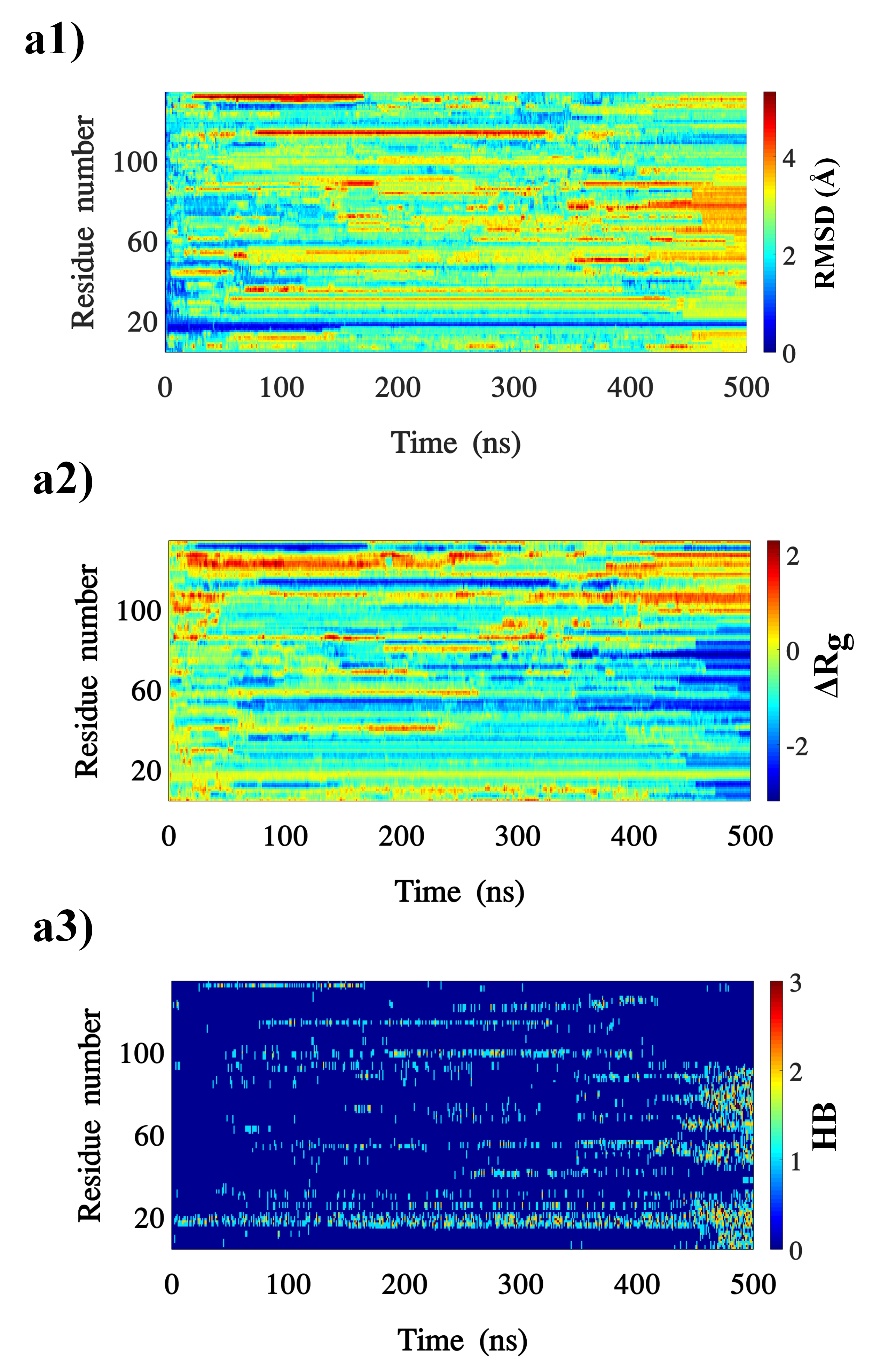


**Fig S2:** The structural properties of protein change during the reverse TMD simulations (extended to helical states). a1) the RMSD of alpha carbons from initial configuration. a2) The difference between gyration radius ($\Delta R_{g}$) of each frame configurations with the first frame during TMD trajectories. a3) The internal hydrogen bond numbers ($HB$) along the simulations. All the properties are calculated over the sliding windows. The locations of the hot sites are colored as red, yellow and cyan in the left side of the plots indicating the first, second and third priorities, respectively.


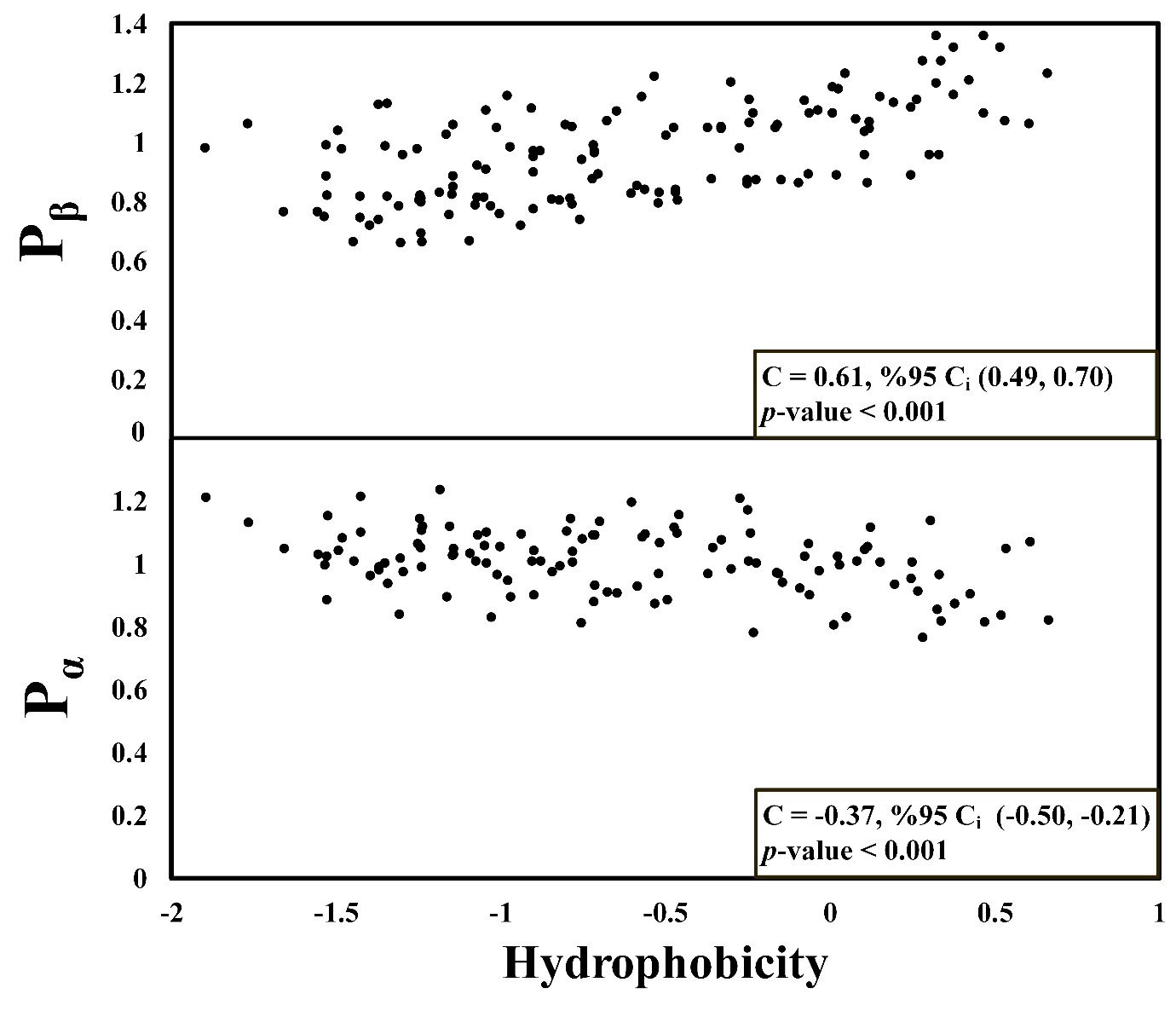


**Fig. S3:** Correlations between hydrophobicity and propensity to extended (top) and helical (bottom) structures. The correlation coefficients in %95 confidence intervals are obtained between P_β_ and P_α_ with hydrophobicity. The p-value is <0.001 for both.


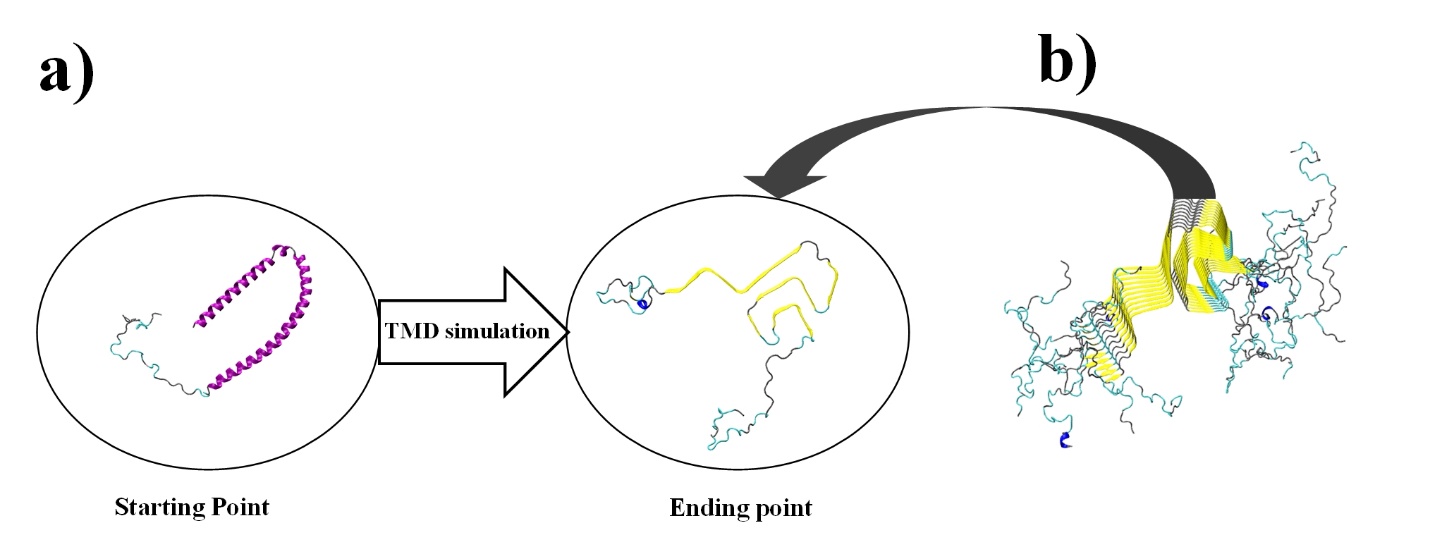


**Fig. S4:** The Starting and ending points of TMD simulations. a) The locally focused on the full-length chains of helical, extended states in αβT. The total amyloid fibril structures are shown in b part. All the structures are colored by secondary structure. Each configuration provided by VMD version 1.9.3 ^62^.


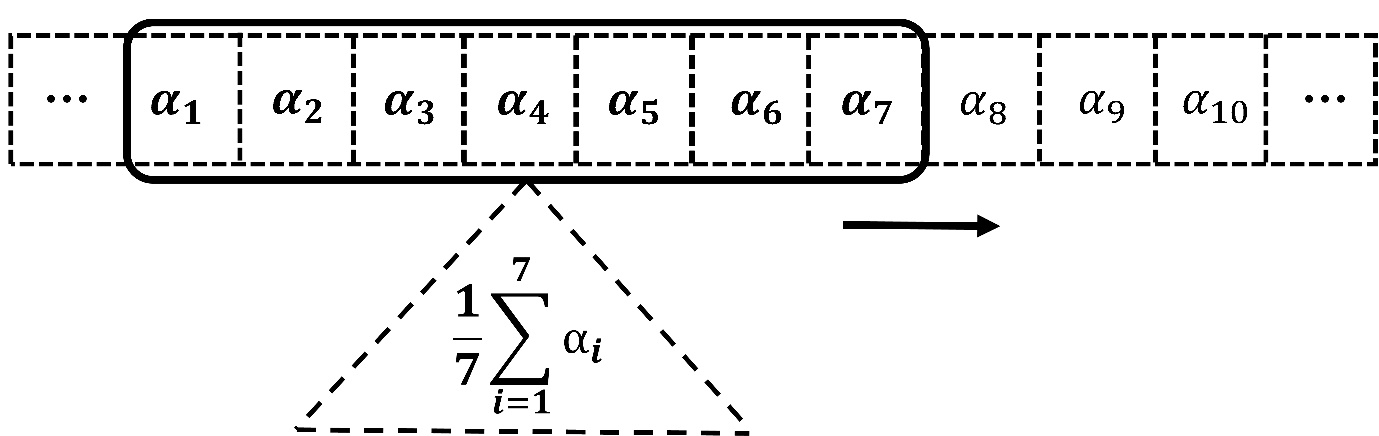


**Fig S5:** The statistical averaging is performed over the sliding windows. The figure shows the averaging of numerical values of $i$th sliding window which is assigned to the $i$th residue. The α is corresponded to value of each residual-dependent property.
